# Maternal Mortality in Colombia in 2011: A Two Level Ecological Study

**DOI:** 10.1371/journal.pone.0118944

**Published:** 2015-03-18

**Authors:** Luz Mery Cárdenas-Cárdenas, Karol Cotes-Cantillo, Pablo Enrique Chaparro-Narváez, Julián Alfredo Fernández-Niño, Angel Paternina-Caicedo, Carlos Castañeda-Orjuela, Fernando De la Hoz-Restrepo

**Affiliations:** 1 Colombian National Health Observatory, Instituto Nacional de Salud, Bogotá, Colombia; 2 Information Center for Public Health Decisions, National Institute of Public Health, Cuernavaca, México; 3 Grupo de Economía de la Salud, Universidad de Cartagena, Cartagena de Índias, Colombia; 4 Instituto Nacional de Salud, Bogotá, Colombia; Columbia University, UNITED STATES

## Abstract

**Objective:**

Maternal mortality reduction is a Millennium Development Goal. In Colombia, there is a large disparity in the maternal mortality ratio (MMR) between and into departments (states) and also between municipalities. We examined socioeconomics variables at the municipal and departmental levels which could be associated to the municipal maternal mortality in Colombia.

**Methods:**

A multilevel ecology study was carried out using different national data sources in Colombia. The outcome variable was the MMR at municipal level in 2011 with multidimensional poverty at municipal and department level as the principal independent variables and other measures of the social and economic characteristics at municipal and departmental level were also considered explicative variables (overall fertility municipal rate, percentage of local rural population, health insurance coverage, per capita territorial participation allocated to the health sector, transparency index and Gini coefficient). The association between MMR and socioeconomic contextual conditions at municipal and departmental level was assessed using a multilevel Poisson regression model.

**Results:**

The MMR in the Colombian municipalities was associated significantly with the multidimensional poverty (relative ratio of MMR: 3.52; CI 95%: 1.09-11.38). This association was stronger in municipalities from departments with the highest poverty (relative ratio of MMR: 7.14; CI 95%: 2.01-25.35). Additionally, the MMR at municipal level was marginally associated with municipally health insurance coverage (relative ratio of MMR: 0.99; CI 95%: 0.98-1.00), and significantly with transparency index at departmental level (relative ratio of MMR: 0.98; CI 95%: 0.97-0.99).

**Conclusion:**

Poverty and transparency in a contextual level were associated with the increase of the municipal MMR in Colombia. The results of this study are useful evidence for informing the public policies discussion and formulation processes with a differential approach.

## Introduction

Maternal mortality (MM) is a widely used indicator of health equality in a population, especially in issues related to gender and women’s health. According to the World Health Organization (WHO), 287 thousands maternal deaths occurred worldwide in 2010 [1]. The WHO also reported that countries in Latin America and the Caribbean had approximately 9,500 maternal deaths in the same year, for a maternal mortality ratio (MMR) of 88.9 per 100,000 live births [1].

Inequality in health care delivery has being hypothesized as an important factor related to the stagnation of MMR and lack of progress in the fifth Millennium Development Goal (MDG) in some countries [2–3]. Colombia, an upper-middle income country, has a Gini index of 0,548 and is considered one of the most unequal countries worldwide [4–5]. According to the 2^nd^ Report of the Colombian National Health Observatory (*Observatorio Nacional de Salud—ONS*), national MMR was 69.3 deaths per 100,000 live births in 2011. Although a decrease has being shown in MMR since 1998, the *ONS* showed that this reduction is not enough to reach the goal of MDG-5 for 2015 [6].

The MMR in Colombia shows a wide variation within regions and departments (states), Bogota, the capital of the country, and states like Quindío, Risaralda and Santander are in track to achieve MDG-5, but Choco and afro-colombian population, has MMR similar to 2010 African countries, like Ethiopia, Ghana, and Ruanda, or similar to Haiti, the country with the highest MMR in Latin America and the Caribbean [6].

Some studies have been identified individual factors like low educational level, and lack of health service affiliation related with an increased risk of MM [7–9]. Important differences had been also found in MM by age, department, urban/rural zone, socioeconomic status and ethnicity [10]. Finally, other aspects like poor access and low quality of health services during attention of pregnancy, delivery, and puerperium, as well as, the inappropriate access to family planning methods, are clearly associated with an increased risk of MM [11].

In general, aspects related to MM at the clinic or individual level of the pregnant women has been studied; however, it is important to consider the premise that causes of individual cases are not necessarily the same causes of the incidence in the population [12], consequently, the differences in the socioeconomic context could likely explain the observed differences in MMR between municipalities and departments.

Globally, different ecologic studies have found an association between socioeconomic characteristics and MMR, for instance, the average of woman’s education, access to water and sanitation, health expenditure per person, coverage of health services, human development index, a corrupt government, and the income inequity [13–17]. In Colombia, a highest MMR at departmental level has been previously correlated with a highest percentage of unsatisfied basic needs (*Necesidades Básicas Insatisfechas NBI*) [18], but the association between MMR and social and economic characteristics in the municipal and departmental level had not been investigated. The aim of this analysis was to identify socioeconomic variables at the municipal and departmental levels associated to the municipal MMR in Colombia.

## Methods

### Ethical Statement

Ethical approval for this analysis was not required because it uses publicly available data. We used measures of the municipalities and departments, information at individual level was not used, and informed consent was not required.

### Study’s design and sample

An ecological study to identify the socioeconomic characteristics of the municipal and departmental context associated to municipal MMR in Colombia during 2011 was carried out. The observation unit was the municipality, 1,094 municipalities were included (97.5% of total 1,122 Colombian municipalities), from all the 33 departments (states) of the country. We excluded municipalities with missed values in a least one variable included into the analysis; four municipalities were excluded because in the 2011 they reported zero live births.

### Information sources

All data of this study were obtained from third parties, different information sources were used. The outcome and independent municipal variables are based on 2011 data, while the independents variables at departmental level were collected over a five-year period (2008–2011) using the most recent available data. Maternal deaths, life births, proportion of rural population, and *Gini* coefficient were obtained from the National Institute of Statistics (*Departamento Administrativo Nacional de Estadística-DANE*) [4, 19–20]. The multidimensional poverty index and territorial participation for the health sector were obtained from National Planning Department (*Departamento Nacional de Planeación-DNP*) [21–22]. Health insurance coverage was obtained from Ministry of Health (*Ministerio de Salud y Protección Social*) [23], and transparency index from Transparency Corporation for Colombia (*Corporación Transparencia por Colombia*) [24].

### Outcome variable

Municipal MMR was the dependent variable; we considered the number of maternal deaths in the numerator and the number of live births in the denominator in 2011 for each municipality. Maternal death was defined according to the International Classification of Diseases, Tenth Revision (ICD-10) [25]. All the maternal deaths in 2011 classified with the following diagnoses were taken into account: Pregnancy with abortion (O00-O08); edema, proteinuria and hypertensive disorders in pregnancy childbirth and the puerperium (O10-O16); other disorders mainly related to pregnancy (O20-O29); maternal care related to the fetus and amniotic cavity and possible delivery problems (O30-O48); complications of labor and delivery (O60-O75); complications predominantly related to the puerperium (O85-O92); other obstetric conditions not elsewhere classified (O95-O99) and causes specified in other chapters (A34, B20-B24, C58, D392, E230, F530-F539, M830). Deaths occurring in other country and with missed information about municipality or time of death were not included.

### Contextual variables

#### Multidimensional poverty

The multidimensional poverty index was the principal independent variable; it was expressed at municipal and departmental level. This index was calculated by the *DNP* with data from the 2010 Quality of Life Survey and 2005 *DANE* population, considering five dimensions of poverty (household educational conditions, children and youth conditions, work, health, and public services access) and fifteen items. The multidimensional poverty index provides a cutoff to identify households deprived in each indicator; a home is multidimensional poor when present at least five deprived items [21].

The multidimensional poverty index estimates two measures: the incidence and the intensity of multidimensional poverty. The incidence of multidimensional poverty is defined as the proportion of people living in multidimensional poor households and it was expressed at municipal and departmental levels. The intensity of multidimensional poverty is the proportion of deprived items among the poors; the intensity of poverty meets monotonicity dimensional feature, i.e., the intensity of poverty increases as the proportion of deprivation among the poor increases; it was only expressed at municipal level [21].

### Covariables

The covariables were grouped into municipalities (first level) and departments (second level). Among the covariables at the municipal level were included: global fertility rate, proportion of rural population, health insurance coverage and per capita territorial participation allocated to the health sector.

The global fertility rate was defined as the average number of children per woman. It was calculated for each municipality following demographic processes of United Nations [26]. The global fertility rate was considered like adjustment variable because it has been understood like a proxy of municipal woman’s reproductive behavior and a predictor of the MM, as has been demonstrated in similar studies [14, 27–29].

The proportion of rural population corresponded to the proportion of municipal people living in rural area, where rural area is defined as the scattered distribution of homes without trace or naming of streets and presence of agricultural exploitation [30].

The health insurance coverage corresponded to the proportion of adult people affiliated to the Colombian public health system (*Sistema General de Seguridad Social en Salud*) and it modality (total or partial subsidized).

Due to Colombia is a decentralized country and each municipality have to ensure services like health, education, safe water and basic sanitation through economic resources that are transferred from the nation to the municipalities and departments, we considered the amount of money in 2011 Colombian pesos (COP) adjusted per capita, which government assigned to each municipality to financing of health services.

As departmental level covariables we included: income inequality expressed in the *Gini* coefficient in the 2011, and the transparency index in 2008, which identifies conditions or practices that promote transparency or, on the contrary, is increasing the risks of corruption in the management of public entities; a high transparency index indicated low risk of corruption [24].

### Statistics analysis

The variables were described by median and interquartile range because it did not have clearly a symmetric distribution. For the analysis, the departmental incidence of multidimensional poverty was categorized into tertiles generating categories: low- (≤ 45.23%), moderate- (45.23% to 61.37%), and high-departmental poverty incidence (> 61.37%).

Considering the hierarchical structure of the municipal data which are grouped into departments, we first estimated the Intraclass Correlation Coefficient (ICC) for the departmental level in a first null model with random intercepts [31]. In this case, ICC can be interpreted as the percentage of variance in the municipal MMR attributable to the departmental level and also as the extent to which municipal level observations are correlated within each department. This first estimation obtained was statistically different to zero (20%; p<0.001), so we consider that multilevel analysis was a first approach. Also considering that the municipalities into a same department tend to share some commons determinants as departmental policies.

Given that the outcome variable was a count, a multilevel Poisson model with random intercepts was adjusted to assess the association between the contextual socioeconomic characteristics and the municipal MM. This model uses an unstructured variance-covariance matrix which was estimated from the analytic sample.

The specification of model was:

$$Ln\left(\mu |\beta o_{j},\;X_{ij}\right)\;=\;\beta o_{j}+\;\beta_{k}x_{i}+\lambda_{i}$$

Where: μ is the expected cases of maternal deaths in each municipality ***i*** of the j department. *λ* is the “exposure” variable with coefficient constrained in 1, which in this context represents a proxy of susceptible population of death which as is widely used in public health is the number of live births for the same municipality. Additionally, *β*_*k*_ *x*_*i*_, is the vector of k independent variables at the municipal level. Alternatively with some arithmetic manipulation, the same model could be written as:

$$Ln\left(\frac{\mu }{\lambda }|\beta o_{j},\;X_{ij}\right)\;=\;\beta o_{j}+\;\beta_{k}x_{i}$$

Where we can see that μ/λ is equivalent to the municipal MMR.

Finally, βoj was the intercept for each j department, it varies randomly between departments, given the model:

$$\beta oj\;=\;\delta o+{\;\delta }_{l}x_{j}+\;Ѱ$$

Where *δo* is the national common intercept and *δ*_*l*_ *x*_*j*_ is the vector of l independent variables at departmental level and *Ѱ* a complex structure of the random error.

Each multilevel model was compared with their equivalent models of fixed effects using the Hausman test [32]. No statistically significant systematic differences in the estimated coefficients are found by comparing these fixed models with random intercepts models (p> 0.10). Therefore, to maintain statistical consistency and not to find a correlation between the independent variables and the random component, we chose random patterns, which are known to be more efficient (have lower variance estimators).

Finally, four models were sequentially adjusted: Model 1 (Null) no independent variables were included; Model 2 (Municipal) only municipal characteristics were included; Model 3 (Departmental) only departmental characteristics were included, and Model 4 (Municipal and Departmental with interactions) was adjusted for municipal and departmental level characteristics and interactions. In the latter, interaction was tested between the municipal incidence of multidimensional poverty and departmental poverty incidence (in tertiles), using a multiplicative term. Separate models were fitted to the municipal intensity of multidimensional poverty in which these interactions were also tested. Fitted models were compared through the Akaike Information Criteria (AIC) [33].

An alpha <0.05 was regarded as statistically significant, except in interactions which it was <0.25. For the results we applied an exponential function to convert them into the relative MMR or the relative ratio of MMR. Enlistment of information was held in MS Excel and analysis in Stata version 12.0 (STATA Corporation, College Station, TX, USA).

## Results

**Fig 1:**
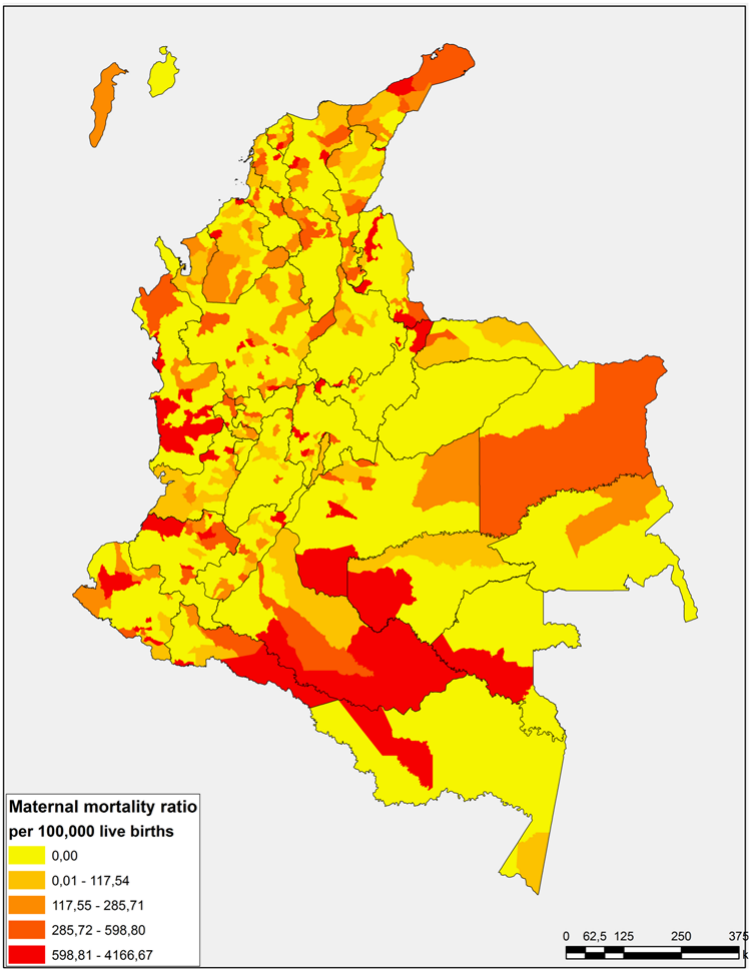
Maternal mortality ratio in Colombian municipalities, 2011.

Twenty two percent of Colombian municipalities reported MM in 2011. The MMR for Colombia during this year was 69 per 100,000 live births, the municipalities with highest MMR were San Miguel and Albania in Santander department and Mangüi in Nariño department with MMR of 4167; 3125 and 2564 per 100,000 live births, respectively. MMR in Colombia was concentrate in municipalities placed in periphery regions of the country like: at South in Amazonas region, at East in Orinoquía region, at West in Pacific region and in the North Coast of Colombia as is shown in Fig. 1. The biggest cities of the country as Bogotá, Cali, Medellin and Barranquilla reported during the same year a high number of cases of MM, however, due to the considerable number of live births to these places, a high MMR was not expressed. With regard to socioeconomic characteristics at municipal and departmental level, half of Colombian municipalities included in this study had more than 70% of its population in situation of multidimensional poverty (Median: 70.71; P_25_-P_75_: 60.44–81.22) but a health insurance coverage upper than 85% (Median: 85.29; P_25_-P_75_: 74.95–95.04). A departmental transparency index upper than 72 was found in 50% of departments (Median: 72.40: P_25–75_: 67.90–78.20), the descriptive statistics from other characteristics at municipal and departmental level are presented in the Table 1.

**Table 1:** Descriptive statistics of characteristics at municipal and departmental level.

Municipal or departmental property	Median (P_25_-P_75_)
**Municipal level**	
Multidimensional municipal poverty (%)	70.71 (60.44–81.22)
Multidimensional poverty intensity (%)	33.84 (27.36–42.06)
Overall fertility municipal rate	1.58 (1.28–1.97)
Percentage of local rural population	60.53 (40.83–75.73)
Health insurance coverage	85.29 (74.95–95.04)
Per capita territorial participation allocated to the health sector (in 2011Colombian pesos)	138,203 (109,342–158,934)
**Department level**	
Multidimensional departmental poverty (%)	55.05 (44.75–68.89)
Transparency index	72.40 (67.90–78.20)
Gini coefficient ^a^	0.53 (0.50–0.54)

P_25:_ 25^th^ Percentil.

P_75:_ 75^th^ Percentil.

^a^ For the Gini coefficient only were available for 23 departments. Worst Gini coefficient for department with missing data was assumed.

Table 2 shows that all selected municipal and departmental characteristics were significantly associated with MMR at the bivariate model except overall fertility municipal rate, and income inequity.

**Table 2:** Bivariate multilevel poisson regression analysis: maternal mortality and municipal and departmental characteristics.

Municipal or departmental property	Relative ratio of MMR (CI 95%)
**Municipal level**	
Multidimensional municipal poverty	7.34 (4.44–12.12) ^£^
Multidimensional poverty intensity	25.46 (12.32–52.62) ^£^
Overall fertility municipal rate	0.95 (0.80–1.13)
Percentage of local rural population	2.40 (1.66–3.46) ^£^
Health insurance coverage	0.99 (0.98–1.00)**
Per capita territorial participation allocated to the health sector (in 2011 Colombian pesos)	
Quartile 1 (≤ $ 109,250)	Reference
Quartile 2 (> $ 109,250 and ≤ $ 138,023)	1.77 (1.35–2.32) ^£^
Quartile3 (> $ 138,023 and ≤ $ 158,955)	1.72 (1.26–2.36)**
Quartile 4 (> $ 158,955)	2.19 (1.62–2.96) ^£^
**Department level**	
Multidimensional departmental poverty
Low	Reference
Moderate	0.96 (0.65–1.41)
High	2.23 (1.56–3.17) ^£^
Transparency index	0.96 (0.95–0.98) ^£^
Gini coefficient ^a^	
Low income inequity (≤0.53)	Reference
High income inequity (>0.53)	1.48 (0.98–2.22)

** p<0.01.

^£^ p<0.001.

MMR: Maternal mortality ratio.

^a^ For the Gini coefficient only were available for 23 departments. Worst Gini coefficient for department with missing data was assumed.

The results of the multilevel Poisson model are presented in Tables 3 and 4. In the Table 3, Null model (model 1), 20% of the variance in MMR at municipal level could be attributed to department level. This variation decreased after adjusting for municipal (model 2) and departmental level characteristics (model 3), being 3% in the model 4 which included two levels’ characteristics and its interactions (model 4). The results of model adjusted by municipal level characteristics only (model 2), showed a MMR significantly associated with the multidimensional municipal poverty incidence (relative ratio of MMR 7.44; CI95%: 2.97–18.67). The contextual characteristics at departmental level associated with MMR were a high multidimensional departmental poverty and the transparency index as presented in the model 3.

**Table 3:** Multilevel poisson regression analysis for municipal maternal mortality in Colombia, 2011.

Municipal or departmental property	Model 1 Null Relative ratio of MMR (CI 95%)	Model 2 Municipal Relative ratio of MMR (CI 95%)	Model 3 Departmental Relative ratio of MMR (CI 95%)	Model 4 Municipal and Departmental with interactions Relative ratio of MMR (CI 95%)
**Municipal level**				
Multidimensional municipal poverty incidence		7.44 (2.97–18.67)^£^		3.52 (1.09–11.38)*
Overall fertility municipal rate		0.96 (0.81–1.15)		0.99 (0.83–1.17)
Percentage of local rural population		0.65 (0.37–1.16)		0.80 (0.45–1.43)
Health insurance coverage		0.99 (0.98–1.00)		0.99 (0.98–1.00)
Per capita territorial participation allocated to the health sector (in 2011 Colombian pesos)				
Quartile 1 (≤ $ 109,250)		Reference		Reference
Quartile 2 (> $ 109,250 and ≤ $ 138,023)		1.19 (0.85–1.65)		1.15 (0.82–1.61)
Quartile3 (> $ 138,023 and ≤ $ 158,955)		1.07 (0.71–1.62)		1.05 (0.69–1.60)
Quartile 4 (> $ 158,955)		1.38 (0.88–2.15)		1.25 (0.79–1.9)
**Department level**				
Multidimensional departmental poverty				
Low			Reference	Reference
Moderate			0.95 (0.67–1.34)	0.75 (0.32–1.75)
High			1.68 (1.16–2.42)**	0.69 (0.28–1.73)
Transparency index			0.98 (0.96–0.99)*	0.98 (0.97–0.99)*
Gini coefficient^a^				
Low income inequity (≤0.53)			Reference	Reference
High income inequity (>0.53)			1.06 (0.81–1.40)	0.97 (0.75–1.27)
Interaction Terms				
MMP x moderate departmental				1.15 (0.25–5.36)
MMP x high departmental				2.03 (0.51–8.04)^ŧ^
**Measures of variation**				
Variance (standard error) of the random component	0.2429 (0.0086)	0.0909 (0.0078)	0.0411 (0.0059)	0.0281 (0.0096)
ICC (%)	20	8	4	3
AIC	1298.914	1255.618	1278.505	1255.549

*p<0.05.

** p<0.01.

^£^ p<0.001.

^ŧ^ p<0.25.

^a^ For the Gini coefficient only were available for 23 departments. Worst Gini coefficient for department with missing data was assumed.

MMR: Maternal mortality ratio.

MMP: Multidimensional municipal poverty.

Moderate departmental: Moderate multidimensional departmental poverty.

High departmental: High multidimensional departmental poverty.

ICC: Intraclass correlation coefficient.

AIC: Akaike information criteria.

**Table 4:** Multilevel poisson regression analysis for municipal maternal mortality in Colombia, 2011.

Municipal or departmental property	Model 1 Null Relative ratio of MMR (CI 95%)	Model 2 Municipal Relative ratio of MMR (CI 95%)	Model 3 Departmental Relative ratio of MMR (CI 95%)	Model 4 Municipal and Departmental with interactions Relative ratio of MMR (CI 95%)
**Municipal level**				
Intensity of multidimensional poverty		28.48 (8.30–97.60)^£^		14.52 (3.70–56.90)^£^
Overall fertility municipal rate	0.96 (0.81–1.14)	0.99 (0.84–1.18)
Percentage of local rural population		0.62 (0.36–1.10)		0.77 (0.44–1.36)
Health insurance coverage		0.99 (0.98–1.00)		0.99 (0.98–0.99)*
Per capita territorial participation allocated to the health sector (in 2011 Colombian pesos)				
Quartile 1 (≤ $ 109,250)		Reference		Reference
Quartile 2 (> $ 109,250 and ≤ $ 138,023)		1.20 (0.88–1.66)		1.14 (0.83–1.58)
Quartile3 (> $ 138,023 and ≤ $ 158,955)		1.07 (0.72–1.60)		1.06 (0.71–1.58)
Quartile 4 (> $ 158,955)		1.35 (0.87–2.07)		1.26 (0.82–1.95)
**Department level**				
Multidimensional departmental poverty				
Low			Reference	Reference
Moderate			0.95 (0.67–1.34)	0.80 (0.59–1.11)
High			1.68 (1.16–2.42)**	1.02 (0.69–1.49)
Transparency index			0.98 (0.96–0.99)*	0.98 (0.96–0.99)*
Gini coefficient^a^				
Low income inequity (≤0.53)			Reference	Reference
High income inequity (>0.53)			1.06 (0.81–1.40)	0.96 (0.74–1.24)
**Measures of variation**				
Variance (standard error) of the random component	0.2429 (0.0086)	0.0578 (0.0082)	0.0411 (0.0059)	0.0239 (0.0097)
ICC (%)	20	5	4	2
AIC	1298.914	1249.6.93	1278.505	1247.88

*p<0.05.

** p<0.01.

^£^ p<0.001.

^a^ For the Gini coefficient only were available for 23 departments. Worst Gini coefficient for department with missing data was assumed.

MMR: Maternal mortality ratio.

ICC: Intraclass correlation coefficient.

AIC: Akaike information criteria.

In Table 3, the model 4, adjusted by municipal and departmental characteristics, shows an association significantly between the multidimensional municipal poverty incidence and the transparency index with the MMR, and a marginally effect for the health insurance coverage (relative ratio of MMR 0.99; CI95%: 0.98–1.00). Additionally, an interaction between the municipal incidence of multidimensional poverty and departmental incidence of multidimensional poverty was found, for each percentual point increase in municipal incidence of multidimensional poverty the relative ratio of MMR was 3.52 (95% CI: 1.09–11.38) in municipalities of low poverty departments, 4.04 (95% CI: 0.86–18.96) in municipalities of moderate poverty departments, and the worst-case scenario was found in the municipalities of departments with high poverty incidence where the relative ratio of MMR was 7.14 (95% CI: 2.01–25.35) times higher for each point increase in the incidence of the municipal multidimensional poverty.

Regarding the MMR and intensity of municipal poverty, the results showed that for every point increase in the proportion of the deprived items between poor people, the relative ratio of MMR was 15.52 times higher (CI95%: 3.70–56.90), adjusting for other independent variables at municipal and departmental level as is presented in the Table 4, model 4. The intensity of multidimensional poverty showed no interaction with departmental incidence of multidimensional poverty.

**Fig 2:**
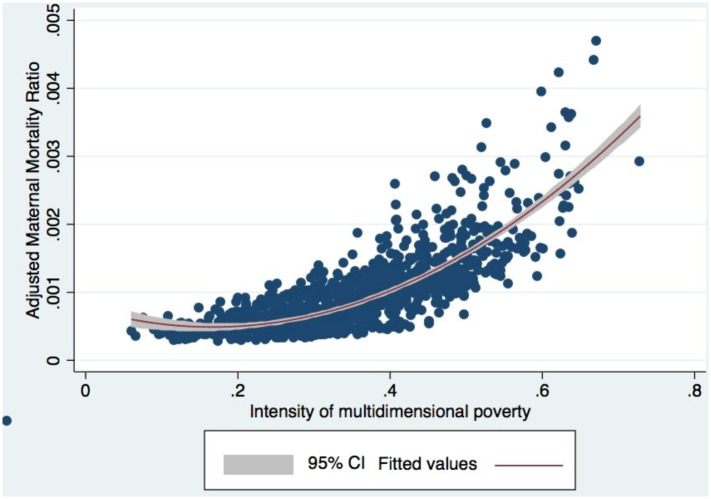
Association between municipal maternal mortality ratio and intensity of multidimensional poverty adjusted by confounding variables.

Finally, Fig. 2 shows the adjusted association between MMR and intensity of municipal poverty.

## Discussion

The main findings of the present study indicate that municipal MMR is significantly associated with the proportion of population living at households multidimensional poor to the municipal level, being that association higher in municipalities of departments with high-department multidimensional poverty. In the same way in the Colombian municipalities the MMR was also associated with the municipal poverty intensity, departmental transparency index, and in a marginal way with municipal health insurance coverage.

The association between municipal and departmental multidimensional poverty and the MMR results in a public health concern, because the 49.6% of the total Colombian population are living in poverty conditions, with more than 80% municipalities with higher values [21]. The effect is very huge when the privations between the poor are higher, indicating that are necessary intersectorial interventions to impact the MM prioritizing poor municipalities of poorest departments.

The association between municipal poverty incidence (construct that includes education, labor, health, children and youth’s conditions, and public services access) and higher MMR is consistent with published national and international evidence. An ecological study in Colombia showed higher MM in departments with higher proportion of unsatisfied basic needs [18]; in other countries like Chile and Iran, there are available evidence for the association of the educational level and the MMR [13–15]. Also, lack to access to public and sanitary services is related to MM in other countries [16].

The possible explication of the association between poverty and MMR is supported in three cornerstones: 1) a low women’s education which decrease skills and practices acquisition with regard to heath care and negatively impacts health services usage rates [14]. In Colombia less probability to meet at least four antenatal controls has been found in women with low education level [5], 2) low per capita income like a barrier to the overcoming of household and sanitation needs and the health services access [16], especially when health care is considered expensive and affect people’s out-of-pocket expenditure, and 3) the effect of lack of basic sanitation in the community on the hygiene of the delivery attention [16].

Although in our study we did not evaluated the association between each poverty items, the strong association found between the multidimensional poverty and the MMR specially in municipalities from poorest departments open many questions because in those municipalities generally in the periphery of the country also concurs other geographical, political, and social issues characterized by rural areas with difficult access, war and conflict, presence of ethnical groups, neglecting by the State to deliver health care [5] and low levels of departmental transparency or high corruption in the public institutions [24]. The coexistence of these issues could likely do an important effect over the women health, taking into account that many of the MMR variability at the municipal level is explained by departmental contextual factors as reported in this analysis.

Another important finding was the association between a high departmental transparency index with a low municipal MMR in coherence with other studies that analyzed the association of corruption (in opposition to the transparency) and the MM at the global level [16]. The transparency index used in the present study account for the operation of the governmental institutions to departmental level in relation to the accountability, procurement management, human resources management, and guarantee the right of access to information, and the index is considered a proxy of governance of the public system [16, 34]. Low transparency index is equivalent to high corruption risk in government institutions and the possibility to affect the health system resource management [16].

In relation to the coverage of the health insurance to the municipal level, the marginally protect association found in this study could indicate us a better access of the woman to health services. Nevertheless, in Colombia some aspects of the health system has been found like underlying conditions in the MM cases, ie., the lack of availability and access of the woman to health services, especially in whom are of the rural area, from any ethnic group and with material privation; the poor quality of service expressed in delayed of referral to a highest complexity service, poor infrastructure, supplies and trained human resources and inefficiency in the promotion and prevention programs reflected in the failure to antenatal care, delivery and postpartum care and limited access to family planning [6], last aspects which are considered in the literature like strategies recommended to MM reduction [14–15, 35].

Although, in our analysis an important role of the poverty over MMR exists, aspects like the availably, access and quality of health services could play a fundamental determinant. Indicators at municipal level of the health service are necessary in Colombia, which could evidence deep inequalities, especially socially marginalized territories [6].

In our study, per capita territorial participation allocated to the health sector was not associated with municipal MMR. Although similar results have been reported in ecological studies worldwide [28], in Colombia it could be argued that not always the resources devoted to health at the municipal level are invested exclusively for the strengthening of induced demand and quality of care programs for women. Under Colombian law these resources should be allocated to cover several aspects: ensuring poor and vulnerable population assurance, provision of health services and implementation of public health plans, giving priority to a wide range of issues not restricted to maternal health. Therefore, future studies should disaggregate the municipal economic resources in health in order to identify the role of these on MMR.

This study has limitations. First, given this is analysis of available data, it was not possible include variables which reflect the pregnant attention to the municipal level like prenatal control program coverage, institutionalized delivery, and postpartum control; aspects that configure system determinants of the MM [14–16, 35]. However, the used regression model was adjusted by variables like proportion of rural population and health insurance coverage, described in the literature as proxies of women health services access [5]. Second, subregistration of MM is also possible, especially in remote areas, however, MM is an event subject of mandatory notification [36]. Third, this analysis has an ecological design and the inference of its findings should realize at the municipal level in order to reduce the possibility of ecological fallacy. In this sense, we cannot ensure that the association is evident at the municipal level reflects an association at the individual level, in this case, the multi-dimensionally poor pregnant women belonging to households have increased risk of MM reported at municipal level.

Strengths of the present study include the national level analysis looking for contextual factors of the municipal and departmental level associated with municipal MMR. The impact of this study in the public health lies in the visualization of gaps in municipal MMR by socioeconomic conditions expressed in different organizational levels (municipal and departmental), conditions like multidimensional poverty and transparency in governmental institutions transcend the Health Sector and need to other social and governmental stakeholders to create strategies to positively impact this problem.

In Colombian context, these structural determinants of MMR allow to national and municipalities authorities to establish the characteristic of the municipalities more vulnerable and building control policies of the MM which transcend the focus of health service delivery. So, the findings here reported are useful evidence for the decision makers and policy makers focused to improve the life conditions, and health in poorest municipalities and departments with differential approach.

Finally, we can conclude that municipal and departmental poverty conditions, as well as, aspects of the health system related to its coverage and governance influence the municipal MMR in Colombia. Results of this analysis reflect the inequality within Colombia, where municipalities with higher poverty level, in poorest departments, and with lower transparency levels have higher MMR comparing with less disadvantages municipalities.
